# Diagnosis of coronary layered plaque by deep learning

**DOI:** 10.1038/s41598-023-29293-6

**Published:** 2023-02-10

**Authors:** Makoto Araki, Sangjoon Park, Akihiro Nakajima, Hang Lee, Jong Chul Ye, Ik-Kyung Jang

**Affiliations:** 1 Cardiology Division, Massachusetts General Hospital, Harvard Medical School, 55 Fruit Street, GRB 800, Boston, MA 02114 USA; 2 Department of Bio and Brain Engineering, Korea Advanced Institute of Science and Technology, 291 Daehak-ro, Daejeon, 34141 South Korea; 3 Biostatistics Center, Massachusetts General Hospital, Harvard Medical School, Boston, MA USA; 4 Division of Cardiology, Kyung Hee University, Seoul, South Korea

**Keywords:** Cardiovascular biology, Interventional cardiology

## Abstract

Healed coronary plaques, morphologically characterized by a layered phenotype, are signs of previous plaque destabilization and healing. Recent optical coherence tomography (OCT) studies demonstrated that layered plaque is associated with higher levels of local and systemic inflammation and rapid plaque progression. However, the diagnosis of layered plaque needs expertise in OCT image analysis and is susceptible to inter-observer variability. We developed a deep learning (DL) model for an accurate diagnosis of layered plaque. A Visual Transformer (ViT)-based DL model that integrates information from adjacent frames emulating the cardiologists who review consecutive OCT frames to make a diagnosis was developed and compared with the standard convolutional neural network (CNN) model. A total of 237,021 cross-sectional OCT images from 581 patients collected from 8 sites were used for training and internal validation, and 65,394 images from 292 patients collected from another site were used for external validation. In the five-fold cross-validation, the ViT-based model provided better performance (area under the curve [AUC]: 0.860; 95% confidence interval [CI]: 0.855–0.866) than the standard CNN-based model (AUC: 0.799; 95% CI: 0.792–0.805). The ViT-based model (AUC: 0.845; 95% CI: 0.837–0.853) also surpassed the standard CNN-based model (AUC: 0.791; 95% CI: 0.782–0.800) in the external validation. The ViT-based DL model can accurately diagnose a layered plaque, which could help risk stratification for cardiac events.

## Introduction

Acute coronary syndromes (ACS) are predominantly caused by plaque rupture or plaque erosion with superimposed occlusive thrombosis^1^. Plaque can destabilize without life-threatening thrombotic luminal occlusion, and most thrombotic lesions remain subclinical, especially when plaque burden is low^2–4^. Over the course of days or weeks, a silently developed thrombus become organized, which results in the formation of a healed plaque, characterized by layers of proteoglycans and type III collagen^5^. In autopsy studies, healed plaques were frequent in men who died of coronary events, with a prevalence of up to 61–73% in whole coronary arteries^6,7^. A histology validation study reported that optical coherence tomography (OCT) can recognize healed plaque as a plaque with one or more layers of different optical densities^8^. In recent in vivo studies, layered plaque at the culprit lesion was associated with higher levels of local and systemic inflammation^9^ and subsequent rapid plaque progression^10^. Identification of patients with layered plaques may help risk stratification for future events. With the early promising results, deep learning (DL) is widely applied to medical imaging due to its potential for automated diagnostic systems^11,12^, and cardiology is not an exception to this trend. Several studies have reported the results of DL application in OCT images^13–15^, but the tasks were limited to the classification or segmentation of plaque characteristics easily discernible by human eyes. In addition, the clinical significance of these studies is limited by the small number of data resulting from the lack of large and well-curated databases for the development of a robust algorithm. Furthermore, previous convolutional neural network (CNN)-based DL models lacked the ability to utilize the information of adjacent OCT frames.

In this study, we developed a vision transformer (ViT)-based model, which has shown remarkable improvement over the CNN-based model in computer vision^16^, to provide the automatic diagnosis of layered plaque using a large and well-curated multi-center database and externally validated in a separate database. The ViT-based model not only utilizes its attention to detect the subtle change in OCT signal in layered plaque but also integrates the information of adjacent OCT frames by an ensemble, emulating the reading process by OCT experts to accurately diagnose layered plaque.

## Methods

### Study design and datasets

Patients presenting with acute coronary syndromes (ACS) who had pre-intervention OCT imaging of the culprit lesion were selected for the training and validation data set from the Predictor study. The Predictor study was an international, multi-center, registry that included ACS patients who underwent OCT at 11 institutions in 6 countries^17^. The data used for the current project was from 8 institutions in 4 countries (Table S1). To externally validate the developed model, patients from the EROSION study were used as a testing data set. The EROSION study was a single-center, prospective study that included ACS patients undergoing OCT and tested the safety of medical therapy instead of stent implantation^18^. Patients with ACS caused by uncommon pathologies such as calcified nodules, spontaneous coronary dissection, or coronary spasm were excluded from this study. The patient selection process is summarized in Fig. S1. The diagnosis of ST-segment elevation myocardial infarction (STEMI) and non-ST-segment elevation acute coronary syndromes (NSTE-ACS) was made according to the concurrent American Heart Association (AHA)/American College of Cardiology (ACC) guidelines^19,20^. Demographic, clinical, and angiographic data were collected at each participating site and the anonymized data were sent to Massachusetts General Hospital (Boston, MA, USA). Details on the definition of the training, internal and external validation datasets are provided in the Supplemental Method. The Predictor study and the EROSION study were approved by the Institutional Review Boards at each participating site (Nara Medical University Hospital Institutional Review Board, Nippon Medical Chiba Hokusoh Hospital Institutional Review Board, Hirosaki University Hospital Institutional Review Board, Partners Human Research Committee, the Chinese University of Hong Kong Institutional Review Board, Tsuchiura Kyodo General Hospital Institutional Review Board, Kitasato University Hospital Institutional Review Board, University Hospitals Leuven Institutional Review Board and the 2nd Affiliated Hospital of Harbin Medical University Institutional Review Board). For the Predictor registry, informed consent was waived by the Institutional Review Boards at each participating site. For the EROSION study, written informed consent was obtained before enrollment. The study protocol conforms to the ethical guidelines of the Declaration of Helsinki.

### Image acquisition and data labeling

The coronary segment that includes the culprit lesion was assessed using a frequency-domain (C7/C8, OCT Intravascular Imaging System, St. Jude Medical, St. Paul, Minnesota) OCT system and a 2.7-Fr OCT imaging catheter (Dragonfly, St. Jude Medical, St. Paul, Minnesota). In the training and internal validation dataset, 12 (2.1%) of patients had 0.25-mm intervals, 372 (64.0%) of patients 0.2-mm intervals, and 197 (33.9%) of patients 0.1-mm intervals. In the external test dataset, 277 (94.9%) of patients had 0.25-mm intervals and 15 (5.1%) 0.2-mm intervals. OCT images were acquired before any percutaneous coronary intervention (PCI) procedures. Aspiration thrombectomy was allowed for occlusive thrombus.

**Figure 1:**
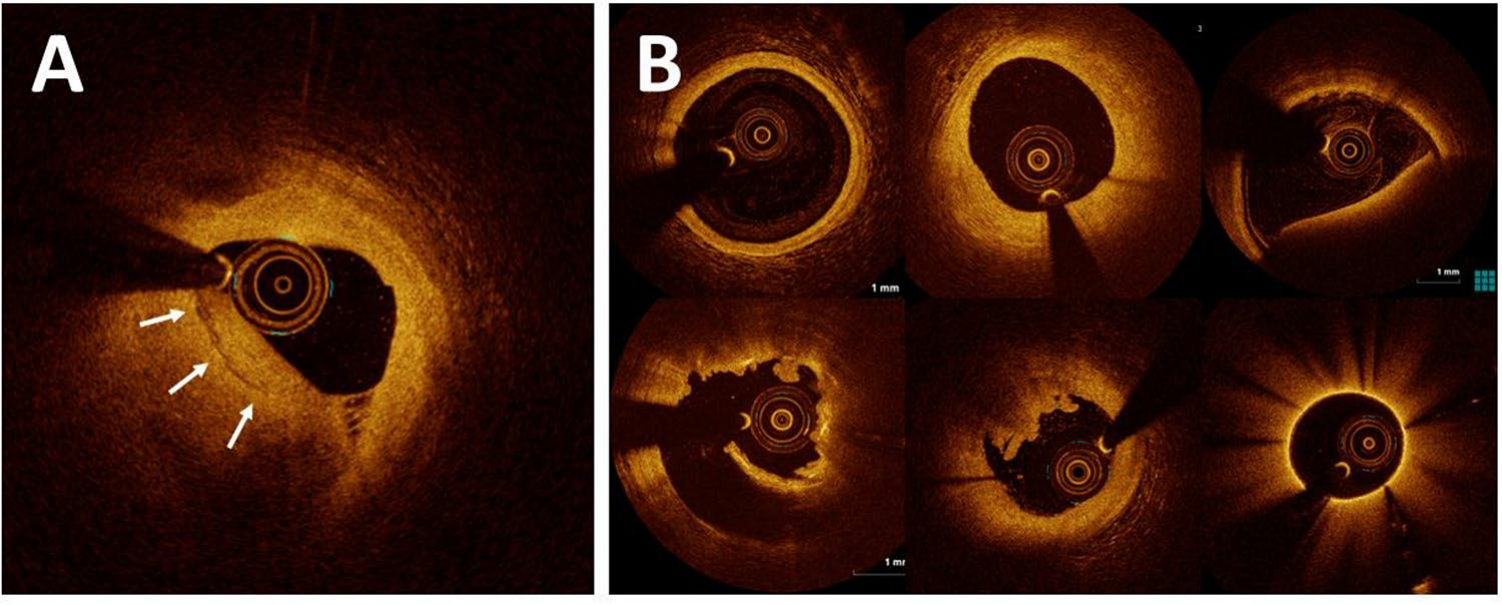
Representative optical coherence tomography image classes. Arrows indicates a layered pattern. OCT frames were classified into two entities: (**A**) layered plaque and (**B**) other in which layered plaque was not observed. Since the automated detection of layered plaque during an OCT pullback is preferred, OCT images also contained sections with normal vessel segment, non-layered plaques, different types of artifacts, and views of guiding catheter.

OCT images were analyzed at the core laboratory at Massachusetts General Hospital. Given that acquiring a definitive label through the collection of a histology sample is impractical for a large number of living patients, a reader with > 8 years of experience in OCT analysis (M.A.) who were blinded to patients’ data independently labeled all OCT images frame-by-frame using an offline review workstation (St. Jude Medical, St. Paul, Minnesota). Layered plaque is defined as a plaque with one or more layers of different optical densities and a clear demarcation from underlying components^21^. Labeling of cross-sectional OCT images in the training, validation, and external testing data sets was done frame-by-frame. Since the automated detection of layered plaque in an OCT pullback is preferred, sections with normal vessel segment, different types of artifacts, and guiding catheter were also included. To develop a DL model for the diagnosis of layered plaque, OCT frames were classified into two entities: (1) layered plaque and (2) other in which layered plaque is not observed (Fig. 1). Anonymized OCT images in DICOM format and their corresponding labels were transferred to the Bio-Imaging, Signal Processing, and Learning Laboratory (BISPL) at Korea Advanced Institute of Science and Technology (KAIST), South Korea for the development and validation of the DL model.

### Development of the deep learning algorithm

**Figure 2:**
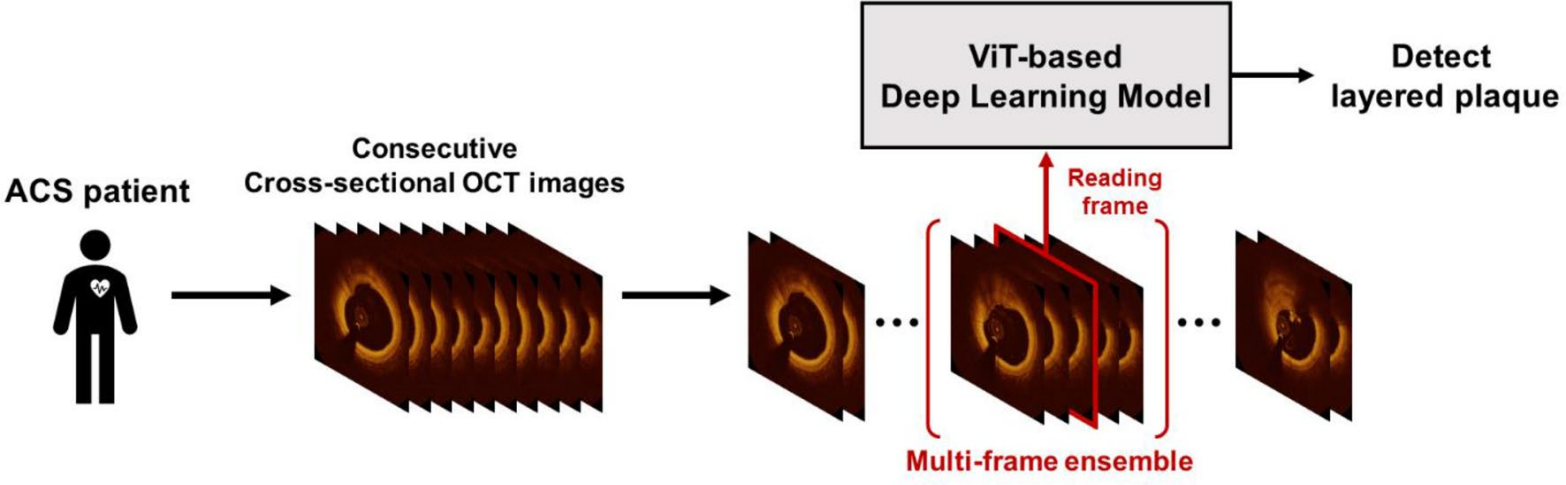
Vision transformer (ViT)-based deep learning model for diagnosis of layered plaque. The proposed ViT-based deep learning model detects the faint sign of layered plaque more accurately than the standard convolutional neural network (CNN)-based deep learning model by maximally utilizing attention mechanism, and further improves its performance via multi-frame ensemble method, which resembles the recognition process of the experienced OCT reader. OCT, optical coherence tomography.

Since the detection of layered plaque is often challenging due to the subtle difference in OCT signal, we sought to develop a DL model tailored for the detection of small changes in OCT images. To diagnose a challenging entity like a layered plaque, OCT experts refer to adjacent frames by playing OCT recording back and forth to be more confident about the reading and devote more attention and time to suspicious findings within a given OCT frame. To emulate this, we adopted the ViT-based model by utilizing the self-attention mechanism^22^ which achieved state-of-the-art performance in computer vision with its inherent strengths of attention mechanism as well as by further enhancing the performance using the multi-frame-based decision with the ensemble of the information from the adjacent frames using a sliding window method (Fig. 2). As the preliminary experiments demonstrated that the multi-frame ensemble consistently boosted the performances (Supplementary Results, Tables S3, S4), all experiments were performed applying this method. For comparison, the standard CNN-based model with similar model complexity (ResNet-34) was used^23^. We trained our DL models with frame-level annotation to enable the detection of layered plaque within a single OCT frame. To yield the optimal performance of the DL models with limited medical imaging data, transfer learning from ImageNet, which is popular in computer vision tasks, was used for both models. For more details on the development of DL algorithms, see the Supplementary Methods. We performed five-fold internal validation to find the best hyperparameters as well as to evaluate model performances in the internal set, and assess the final performance in the external validation with a totally different patient group to evaluate the generalization capacity of the model. To provide the interpretation of the models’ decisions, the visualization methods were used to give a transparent interpretation of the model attention. For the ViT-based model, a direct attention map was obtained as it is feasible to directly visualize the self-attention. On the other hand, the indirect attention map was visualized with Grad-CAM-based saliency map for the standard CNN-based model^24^, since it does not use the self-attention mechanism. Attention visualization methods are detailed in the Supplementary Methods.

### Statistical analysis

Continuous variables with a normal distribution were expressed as mean ± standard deviation (SD), while the median (interquartile range) was used to summarize non-normally distributed variables. Normally distributed variables were compared using the Student’s *t* test and non-normally distributed variables were compared using the Mann–Whitney *U* test. Categorical data were expressed as absolute frequencies and percentages, and compared using the Chi-square test or Fisher exact test, as appropriate.

To evaluate the model performance, the area under the receiver-operating-characteristic curve (AUC) was calculated with 95% confidence intervals (CIs) using the DeLong test^25^. Likewise, sensitivities, specificities, accuracy, false-positive rate (FPR), and false-negative rate (FNR) were obtained with 95% CIs using the “exact” Clopper-Pearson confidence intervals. For model comparison, AUC was used as the performance metric and compared between models with the DeLong test to determine statistical significance. All analyses were performed using R software version 3.6.2 (R Foundation for Statistical Computing, Vienna, Austria) and the Python library scikit-learn version 1.3.3.

## Results

### Study population

For the development and internal validation of the model, a total of 237,021 cross-sectional OCT images collected from 581 patients from 8 institutions in 4 countries were used. For the cross-validation, the 581 patients were randomly divided five-fold into training (n = 465) and internal validation (n = 116) datasets. The developed DL models were further evaluated in another database containing a total of 65,394 images collected from 292 patients from another institution. Patient and lesion characteristics of training, internal validation, and external validation datasets are described in Table 1. The median number of OCT frames for each patient was 375 (range, 217– 540) for the training and internal validation dataset and 217 (range, 206–374) for the external validation dataset. In the training and internal validation dataset, 159 (27.4%) patients had layered plaque, whereas 112 (38.4%) patients had layered plaque in the external validation dataset. At the frame-level, 3894 (1.6%) frames were classified as layered plaque in the training and internal validation dataset, and 1984 (3.0%) were classified as layered plaque in the external validation dataset.

**Table 1:** Patient and lesion characteristics.

	Total	Training and validation	External testing
Number of patients	873	581	292
Demographic data			
Age, years	61.9 ± 12.1	64.4 ± 12.2	56.9 ± 10.4
Male	686 (78.6)	464 (79.9)	222 (76.0)
BMI, kg/m^2^	25.1 ± 3.6	25.0 ± 4.1	25.2 ± 2.7
Hypertension	492 (56.4)	358 (61.6)	134 (45.9)
Hyperlipidemia	456 (52.2)	437 (75.2)	19 (6.5)
Diabetes mellitus	254 (29.1)	187 (32.2)	67 (22.9)
Current smoking	435 (49.8)	231 (39.8)	204 (69.9)
Renal insufficiency	114 (13.1)	106 (18.2)	8 (2.7)
Previous MI	42 (4.8)	34 (5.9)	8 (2.7)
Previous PCI	46 (5.3)	41 (7.1)	5 (1.7)
Previous CABG	3 (0.3)	1 (0.2)	2 (0.7)
Clinical presentation			
STEMI	653 (74.8)	366 (63.0)	287 (98.3)
NSTE-ACS	220 (25.2)	215(37.0)	5(1.7)
Laboratory data			
Total cholesterol, mg/dl	189.5 ± 41.2	192.5 ± 40.7	183.2 ± 41.5
LDL-C, mg/dl	127.2 ± 40.4	128.5 ± 41.4	124.2 ± 37.8
HDL-C, mg/dl	46.5 ± 11.5	46.2 ± 11.6	47.4 ± 11.3
Triglycerides, mg/dl	110.8 (67.0–161.3)	100.0 (58.0–150.0)	136.4 (96.6–185.6)
HbA1c, %	6.4 ± 1.3	6.3 ± 1.3	6.5 ± 1.5
Creatinine, mg/dl	0.87 ± 0.48	0.93 ± 0.57	0.75 ± 0.19
Hs-CRP, mg/dl	0.34 (0.10–4.76)	0.10 (0.05–0.30)	7.15 (3.24–12.48)
Peak CK, IU/l	1517.0 (413.8–2995.3)	1136.0 (270.0–2917.3)	1997.5 (1083.3–3079.5)
Peak CK-MB, IU/l	160.9 (47.8–296.6)	132.3 (32.0–291.0)	182.3 (86.7–313.0)
LVEF, %	56.1 ± 10.2	55.7 ± 11.4	56.8 ± 8.0
Angiographic data			
Infarct-related artery			
RCA	320 (36.7)	207 (35.6)	113 (38.7)
LAD	458 (52.5)	305 (52.5)	153 (52.4)
LCx	95 (10.9)	69 (11.9)	26 (8.9)
Minimum lumen diameter, mm	0.64 ± 0.63	0.42 ± 0.57	1.08 ± 0.49
Reference vessel diameter, mm	3.02 ± 0.68	2.88 ± 0.70	3.31 ± 0.52
Lesion length, mm	15.9 ± 7.5	15.5 ± 6.9	16.7 ± 8.6
Diameter stenosis, %	79.7 ± 18.4	86.1 ± 17.1	67.2 ± 14.0
OCT findings			
Pathobiology			
Plaque erosion	337 (38.6)	251 (43.2)	86 (29.5)
Plaque rupture	536 (61.4)	330 (56.8)	206 (70.5)
Lipid-rich plaque	647 (74.1)	389 (67.0)	258 (88.4)
Thin-cap fibroatheroma	383 (43.9)	199 (34.3)	184 (63.0)
Macrophage	629 (72.1)	401 (69.0)	228 (78.1)
Cholesterol crystal	222 (25.4)	138 (23.8)	84 (28.8)
Calcification	296 (33.9)	236 (40.6)	60 (20.5)
Layered plaque	271 (31.0%)	159 (27.4%)	112 (38.4%)

Values shown are n (%), mean ± standard deviation, or median (25th–75th percentile). *BMI* body mass index, *CABG* coronary artery bypass graft, *CK* creatine kinase, *CK-MB* creatine kinase-MB, *HbA1c* hemoglobin A1c, *HDL-C* high-density lipoprotein-cholesterol, *Hs-CRP* high-sensitivity C-reactive protein, *LAD* left anterior descending artery, *LCx* left circumflex artery, *LDL-C* low-density lipoprotein-cholesterol, *LVEF* left ventricular ejection fraction, *MI* myocardial infarction, *NSTE-ACS* non-ST-segment elevation acute coronary syndrome, *PCI* percutaneous coronary intervention, *RCA* right coronary artery, *STEMI* ST-segment elevation myocardial infarction.

### Model performances in the five-fold cross-validation and the external validation

**Figure 3:**
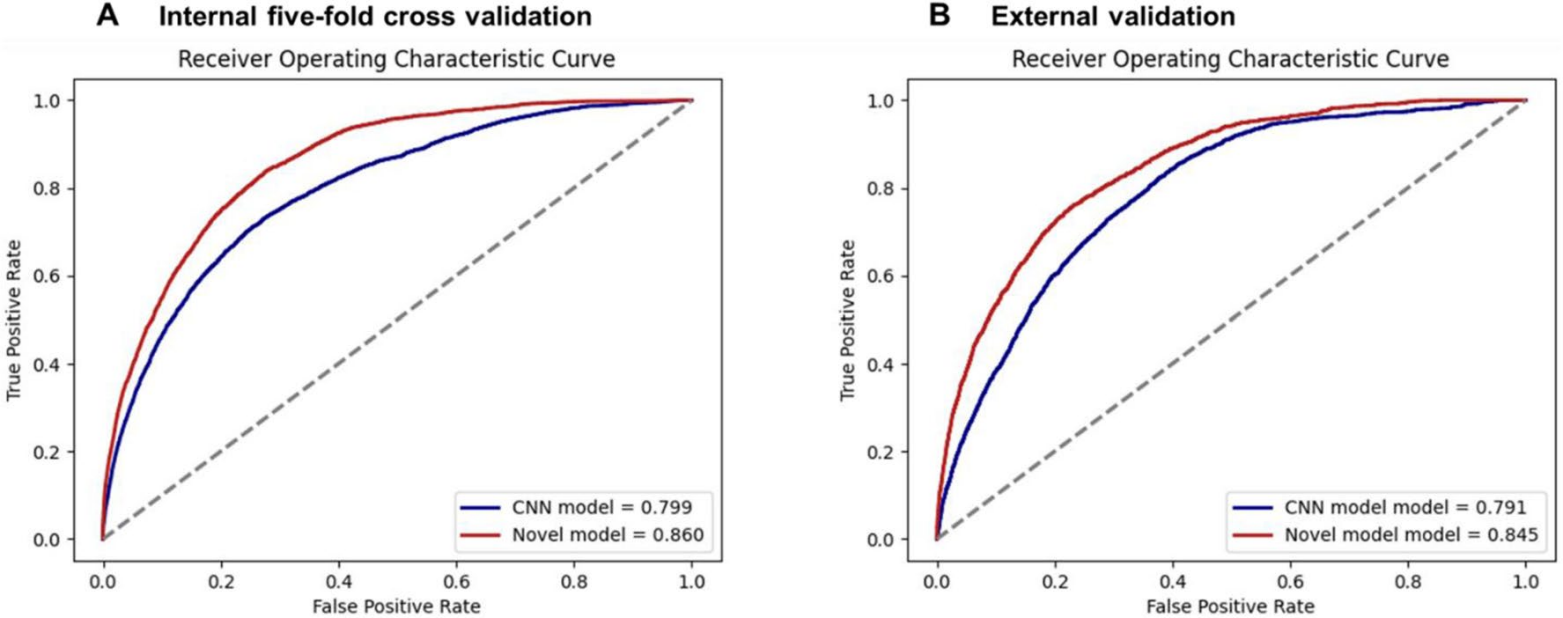
Diagnostic performances of deep learning models in the internal five-fold cross validation and external validation. (**A**) In the internal five-fold cross validation, the vision transformer (ViT)-based model significantly outperformed the convolutional neural network (CNN)-based model (p < 0.001). (**B**) In the external validation, the ViT-based model also showed significantly better performance than the CNN-based model (p < 0.001), showing better generalizability in unseen data distribution.

Diagnostic performance for layered plaque in the five-fold cross-validation is shown in Fig. 3 and Table 2. In the internal five-fold cross-validation, the ViT-based model showed significantly improved performances (p < 0.001) with an AUC of 0.860 (95% CI 0.855–0.866), a sensitivity of 77.7% (95% CI 76.4–79.0), specificity of 77.6% (95% CI 77.4–77.8) and accuracy of 77.6% (95% CI 77.4–77.8) to detect layered plaque within a given OCT frame, compared to those of 0.799 (95% CI 0.792–0.805), 71.7% (95% CI 70.2–74.0) and 73.8% (95% CI 73.6–74.0), 73.8% (95% CI 73.6–73.9) for the standard CNN-based model. The improved performances with the ViT-based model over the standard CNN-based model were maintained with the statistical significance (p < 0.001) in the external validation, showing the AUC of 0.845 (95% CI 0.837–0.853), the sensitivity of 76.5% (95% CI 74.6–78.4), specificity of 76.0% (95% CI 75.7–76.3), and accuracy of 76.0% (95% CI 75.7–76.3) compared to those with 0.791 (95% CI 0.782–0.800), 71.4% (95% CI 69.3–73.4), 71.9% (95% CI 71.6–72.3), and 71.9% (95% CI 71.6–72.2) of the standard CNN-based model. After adapting the model for the patient-level diagnosis, the diagnostic performances at the patient-level are provided in Supplementary Results and Fig. S5, Table S5.

**Table 2:** Performance of the deep learning models for frame-level diagnosis in the internal and external validation.

	AUC (95% CI)	Sensitivity (%) (95% CI)	Specificity (%) (95% CI)	Accuracy (%) (95% CI)
Internal validation				
ViT-based model	0.860 (0.855—0.866)	77.7 (76.4–79.0)	77.6 (77.4–77.8)	77.6 (77.4–77.8)
Standard CNN-based model	0.799 (0.792–0.805)	71.7 (70.2–74.0)	73.8 (73.6–74.0)	73.8 (73.6–73.9)
External validation				
ViT-based model	0.845 (0.837–0.853)	76.5 (74.6–78.4)	76.0 (75.7–76.3)	76.0 (75.7–76.3)
Standard CNN-based model	0.791 (0.782–0.800)	71.4 (69.3–73.4)	71.9 (71.6–72.3)	71.9 (71.6–72.2)

*AUC* area under the curve, *CI* confidence interval, *PPV* positive predictive value, *NPV* negative predictive value, *DL* deep learning, *ViT* vision transformer, *CNN* convolutional neural network.

### Analysis of the false estimate rates (Table 3)

**Table 3:** Performance of the deep learning models in the false-positive rate and false-negative rate analysis.

	False-positive rate (%) (95% CI)	False-negative rate (%) (95% CI)
Internal validation		
ViT-based model	22.3 (21.0–23.6)	22.4 (22.2–22.6)
Standard CNN-based model	28.3 (26.0–29.9)	26.2 (26.0–26.4)
External validation		
ViT-based model	23.5 (21.6–25.4)	24.0 (23.7–24.3)
Standard CNN-based model	28.6 (26.6–30.7)	28.1 (27.7–28.4)

*FPR* false-positive rate, *CI* confidence interval, *FNR* false-negative rate, *DL* deep learning, *ViT* vision transformer, *CNN* convolutional neural network.

In the internal five-fold cross-validation, the FPR and FNR of the ViT-based model were 22.4% (95% CI 22.2–22.6) and 22.3% (95% CI 21.0–23.6), which was lower than those of 26.2% (95% CI 26.0–26.4) and 28.3% (95% CI 26.0–29.9) for the standard CNN-based model. In the external validation, the ViT-based model exhibited FPR and FNR of 24.0% (95% CI 23.7–24.3) and 23.5% (95% CI 21.6–25.3), while those of the standard CNN model was 28.1% (95% CI 27.7–28.4) and 28.6% (95% CI 26.6–30.7).

### Model interpretability

**Figure 4:**
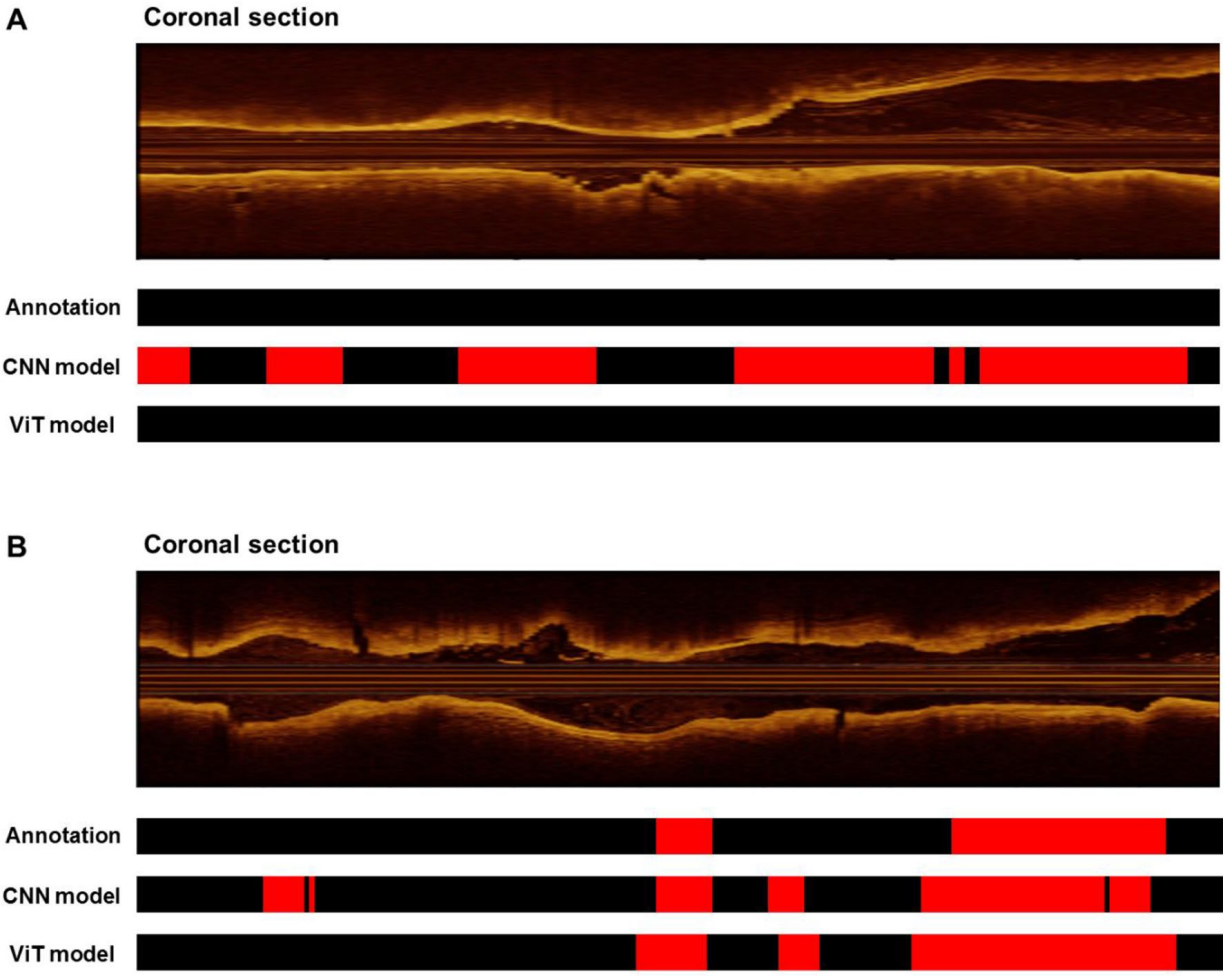
Visualization of the model predictions in sequence-level. As shown in the representative cases, the vision transformer (ViT)-based model showed higher agreement with the ground truth annotation than the convolutional neural network (CNN)-based model, both in (**A**) the negative case and (**B**) the positive case for layered plaque.

**Figure 5:**
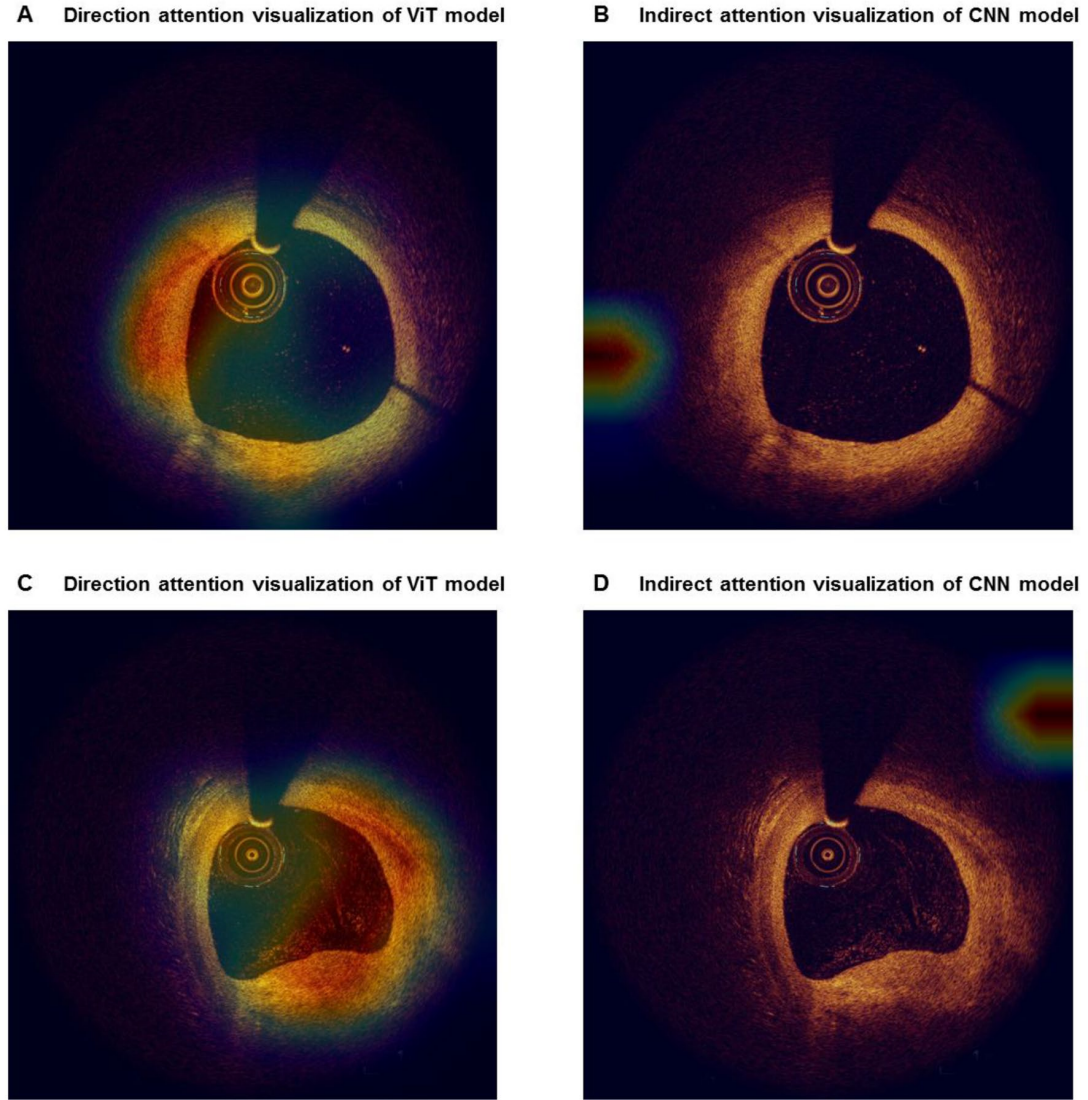
Visualization of the attention of deep learning models. (**A**,**C**) The direct visualization of multi-head attention of vision transformer (ViT)-based model more accurately locate the target lesion than (**B**,**D**) the indirect visualization of network attention via gradient-weighted class activation map in convolutional neural network (CNN)-based model.

To provide a better interpretation of the attention by DL models, we visualized the models in two ways. We first visualized the prediction of two DL models and compared them to the ground truth annotation (label), for both positive and negative cases of layered plaque. As shown in the representative cases in Fig. 4, the ViT-based model showed a high correlation with the ground truth annotation in both positive and negative cases, while the standard CNN-based model did not. As shown in Fig. 5, the attention within a given frame provides the interpretation of the model’s decision by localizing the most “attended” area within the OCT image. The ViT-based model located the layered plaques in the representative case, suggesting that the novel model may “attend” more properly on clinically relevant features. The standard CNN-based model showed inappropriate attention to the background signal suggesting the sign of overfitting^26^.

## Discussion

In this study, we developed and evaluated the deep learning models for the diagnosis of layered plaque, which often exhibits a subtle OCT signal change in the plaque. To this end, a ViT-based model leveraging the self-attention mechanism as well as the multi-frame ensemble was devised, which resembles the reading process of the experienced OCT reader. The experimental results suggest that the ViT-based model can accurately diagnose the layered plaque with AUC over 0.850, which is difficult even for a human reader without years of experience in OCT image interpretation, outperforming the standard CNN-based model in both internal five-fold cross-validation and external validation (p < 0.001 for both).

Pathology studies have shown that atherosclerotic plaques frequently destabilize without clinical consequences^6,7^. Whether an ACS develops following disruption of a plaque depends on the severity of stenosis, and the balance between systemic/local thrombogenicity and endogenous anti-thrombotic/thrombolysis mechanisms. Thrombus becomes organized, with connective tissue deposition of predominantly proteoglycans and types III collagen^21,27^. During the healing process, type III collagen is gradually replaced by type I collagen, which appears as a band of high backscattering signal on OCT^21,27^. Layered plaque has been reported to be associated with multivessel coronary disease, complex lesions, and vulnerable plaque features^9,28^. In a recent study, layered plaque was reported to be a predictor of subsequent plaque progression^10,29^.

When interventional cardiologists diagnose a layered plaque, layered pattern is sought by visual inspection. This approach is subjective and involves the risk of inter-observer variability. Indeed, inter-observer kappa values were not excellent (0.73–0.78) in previous studies^10,28,30,31^. Therefore, we devised the DL algorithm for the objective diagnosis of layered plaque. Until now, only a few studies have reported the results of the application of deep learning to the diagnosis of specific OCT findings. Although Min et al. have reported the deep learning model for the diagnosis of thin-cap fibroatheroma^15^, the results were drawn from a small single-center database without external validation. Likewise, Avital et al. developed a standard CNN-based deep learning model for the identification of coronary calcification^32^; however, they used a small number of data with only 8000 and 1500 frames for the model development and the evaluation, respectively. Of note, these two datasets were derived from the same population. In contrast, we utilized a large, well-curated database collected from multiple institutions. The performance of the developed model was evaluated in a separate database collected from another institution to ensure that the model works stably in populations with different characteristics. Our model retained the excellent performance with AUC around 0.850 in the external validation.

With its early success, CNN has been recognized as the de-facto DL model in classification, detection, and segmentation tasks in medical imaging^33^. However, CNN may not be optimal for processing OCT images because the sequential structure within OCT frames cannot be modeled. When OCT experts diagnose a layered plaque, they need to assess a set of OCT frames as a whole. Because of this complex recognition process, the standard CNN-based model, which utilizes the selected frame only, failed to show optimal performance for the diagnosis of layered plaque. Instead, we adopted the recently developed deep learning model, named Vision Transformer (ViT)^16^, which can find important areas in an image with an attention mechanism and has rapidly become a state-of-the-art model in many computer vision tasks. We adapted this model to pinpoint the indistinct signal change of layered plaque in OCT images and utilize the global relationship between adjacent OCT frames. Our results indeed demonstrated that the ViT-based model can diagnose OCT findings more accurately compared with the standard CNN-based model.

Interestingly, DL models could differentiate layered plaque from the normal vessel wall with a three-layer structure (intima, media, and adventitia) (Fig. 5). Furthermore, thanks to the attention mechanism, the ViT-based model could point out the location of layered patterns more accurately compared with the CNN-based model. The suspected lesion locations can be annotated on real-time OCT images to assist cardiologists in making an accurate diagnosis. The viT-based model can also be applied in medical imaging which requires the review of sequential images such as intravascular ultrasound, computed tomography, or magnetic resonance imaging.

### Study limitations

Our study has some limitations. First, interpretation by an experienced reader was used as the ground truth rather than histology validation. This approach was taken, as it was impossible to use histology validation for the development of a new deep learning model using intracoronary imaging. Histology validation rather than interpretations by OCT experts would be ideal as the ground truth. However, obtaining a large number of cases is vital for the development of a DL model, which is not feasible in histology validation studies. Second, the decision to perform OCT was left at the discretion of each operator, although OCT was routinely used at the participating institutions. Patients with poor OCT image quality were excluded. Therefore, selection bias cannot be excluded. Third, although this is the largest study so far for the development of DL models for OCT, the number of positive cases of layered plaque is relatively small and still not sufficient to generalize the results of the current study. Fourth, although the final performance was assessed in the external validation dataset with a totally different patient group to evaluate the generalization capacity of the model, labeling of OCT images in the external validation dataset was done by the same reader as in the training and validation datasets. Therefore, it is possible that the deep learning model has learned reader-specific habits. Fifth, the intra-patient clustering within the OCT volumes might have decreased the standard error estimates and therefore increased the possibility of the type 1 error, which may result in decreased range of the CIs. Sixth, the indirect attention map was visualized with a Grad-CAM-based saliency map for the standard CNN-based model, while a direct attention map was obtained for the ViT-based model. Hence, it is difficult to compare the saliency results directly.

## Conclusions

In this study, we demonstrated that the ViT-based model, which has the attention mechanism, can accurately diagnose layered plaque, outperforming the standard CNN-based model. Further studies that evaluate the possible application of this novel diagnostic model in clinical practice may facilitate the widespread utilization of OCT for the diagnosis of layered plaque.

## Supplementary Information

Supplementary Information.

## Data Availability

The datasets used and/or analyzed during the current study are available from the corresponding author on reasonable request.
