# Supplementary material for: Diagnosis of coronary layered plaque by deep learning
Source: Sci Rep. 2023 Feb 10;13:2432. doi: 10.1038/s41598-023-29293-6 (PMC9918456; doi:10.1038/s41598-023-29293-6)
Supplement: Supplementary file 1 — Supplementary Information. [file 41598_2023_29293_MOESM1_ESM.docx]

**Supplementary Methods**

**Definition of the training, internal validation, and external validation datasets**

In deep learning (DL), data is generally divided into two major datasets: “training” and “validation” datasets. These datasets are independent of each other and do not overlap. The primary training data set is also referred to as the “training and internal validation” dataset, as it can be further split into “training” and “validation” datasets. The training dataset is a data set used to train the weights of the network. The deep neural network generally receives subsets called “batch” from the training data set and optimizes its weights via backpropagation. In general, the training is performed by repeating this optimization process until the network finds the optimal weight parameters. As the deep neural network usually overfits the training data during the training, the internal validation dataset is used for this account to determine the hyperparameter such as the proper stopping point to prevent overfitting. The external validation dataset is a set used to evaluate the performance of the deep neural network trained and tuned with primary training and internal validation dataset, which contain data obtained at different institutions, usually with different devices and clinical settings. This will ensure how well the network will be generalized in a different dataset, which is related to the clinical applicability of the system. In the current study, we used the Predictor study data, which is from 8 institutions in 4 countries, as the primary training and internal validation dataset, and the EROSION study data, which is from a single institution, as the external validation dataset.

**Development of the Deep Learning Algorithms**

To develop a tailored deep learning (DL) algorithm to process OCT images, we devised a novel DL model based on Vision Transformer (ViT) and further enhanced its performance with an ensemble of adjacent frames, which resembles the recognition process of an experienced OCT reader, who “attend” to the suspicious lesion within a given OCT frame and make final diagnosis considering what was seen in the adjacent frames (usually 2-3 frames) before and after the reading frame. For comparison, the standard convolutional neural network (CNN)-based model with a similar model complexity was also implemented (21.8 million parameters for the ViT-based model and 21.9 million parameters for the CNN-based model, respectively).

Detailed descriptions of the DL models are provided in Figure S2. For the ViT-based model, we implemented the ViT model proposed by (1), which divides an image into a number of 16 $\times$ 16 patches and has 12 layers of transformer encoders equipped with 6 heads per layer. The output of the ViT model was a feature vector with a dimension of 384, and this feature vector was then subsequently used as the input of 2 layered multi-layer perceptron (MLP) to yield the final prediction for each OCT frame. As the previous studies have suggested that the ViT can automatically learn robust semantic information via self-supervised learning without the label, the ViT model was first pre-trained in the ImageNet dataset (2) with the self-supervised learning method proposed in (3), and then fine-tuned to the downstream task of diagnosing layered plaque. For the standard CNN-based model, ResNet-34 (4) model was implemented. Since the hidden dimension of the final feature vector was 512, this feature vector was utilized as input for the MLP the same as in the ViT-based model. The CNN-based model also underwent pre-training, but with supervised learning, in the ImageNet dataset and fine-tuned to the downstream task similar to the ViT-based model.

For pre-processing the input OCT images were first resized to 224 $\times$ 224 to maximally deploy the ImageNet pre-trained weights and to reduce the memory usage of DL models. Various data augmentation methods (rotation, shearing, scale, and flip) have been applied to enrich the data variability of the training data. The best hyperparameters were searched during the internal validation, and the details about the hyperparameters for each model were provided in Table S2.

As the dataset contains extremely imbalanced data for each label (233,129 frames for non-layered plaque and 3,894 frames for layered plaque in the training and validation data), we adjusted weights for each class to alleviate the problem of data imbalance during the training and validation, by the weighted cross-entropy loss function defined as below,

$$Loss=-\Sigma weight\left[ class \right]y\left[ class \right]\log\left( p[class] \right)$$

where *weight[class]* is the class weight, *y[class]* is the label for the class, and *p[class]* is the predicted probability for the class. The weights for each class were calculated to be inversely proportional to the number of data for each label. By this, the effect on the network parameters by major and minor classes can be balanced by giving a larger weight to the minor class while giving smaller weights to the major class. Similarly, additional weights were used to satisfy the pre-defined sensitivity ($>$ 0.80) during inference, defined as below,

$$P_{weighted}\left[ class \right]=\frac{weight\left[ class \right]p[class]}{\Sigma weight\left[ class \right]p[class]}$$

where *P _weighted_[class]* stands for the weighted probability for the class, *weight[class]* is the class weight, and *p[class]* is the predicted probability for the class by the model.

**Multi-frame ensemble**

When experienced OCT readers diagnose a specific disease or finding on each OCT frame, they usually go through OCT recordings back and forth to be more confident about their diagnosis. This procedure can be practically helpful to prevent the wrong diagnosis, by correcting confusing diagnoses based on the findings of adjacent frames. Therefore, to emulate this recognition process, we devised a multi-frame ensemble method to incorporate the information from adjacent frames as depicted in Figure S3. Specifically, the model outputs for each frame, which stand for the probabilities of the presence of layered plaque within each frame, were averaged over adjacent 5 frames (window size of 5 frames) as described below,

$$p_{\mathrm{ensemble}}=\frac{{(p}_{n-2}+p_{n-1}+p_{n}+p_{n+1}+p_{n+2})}{5}$$

where $p_{\mathrm{ensemble}}$ is the ensembled probabilities for $n$^th^ frame, and $p_{n}$ denotes model output probabilities for $n$^th^ frame. By this ensemble, it is possible to correct the discontinuous error as the representative cases exemplified in Figure S4.

As this method requires two frames before and after the frame of interest, it was infeasible to apply the method to the first and the last two frames of the given OCT volume. As the first and the last two frames do not include clinically meaningful images (in all patients, they include the frames with either the catheter artifact or the normal image), the model’s prediction for those frames was not utilized.

**Attention Visualization Methods**

Providing interpretations on the model’s decision is of paramount importance in deep learning application on medical imaging, as it can help the physician to determine whether or not the model yield the output based on the proper feature. Therefore, the interpretable deep learning models have recently been gaining attention. Visualizing the attention of the model is one of the popular methods for model interpretability in deep learning. Among these, the gradient-weighted class activation map (Grad-CAM) can faithfully highlight the important area in an image for model prediction for image classification (5). In addition, this method can be applied in any type of model even in the case of the model that does not utilize direct attention like the conventional CNN-based model by indirectly visualizing the most important area for classification. We consequently used this method for attention visualization of the standard CNN-based model (ResNet-34) in our experiment. On the other hand, for the model leveraging the attention mechanism such as ViT, directly visualizing the weights of multi-head attention offers excellent results as well as is more straightforward. For this reason, we directly visualized the attention of multi-heads in the last layer of the transformer encoder within our ViT-based model. The concept of these attention visualization methods for each model is depicted in Figure S4.

**Supplementary Results**

**Comparison of performances with and without multi-frame ensembling**

Table S3 and S4 show the comparisons of the model performances with and without the multi-frame ensembling method. The performances of both the ViT-based model and the CNN-based models are consistently boosted when the multi-frame ensemble was applied. Therefore, we have applied the multi-frame ensembling to all experiments for both ViT-based and CNN-based models.

**Patient-level analysis**

Since the model that can provide the patient-level prediction results of the layered plaque may have useful clinical applicability, we have adapted our ViT-based model for the patient-level diagnosis as suggested in Figure S5. In detail, the model integrates the frame-level features and makes the diagnosis for the entire OCT volumes of each patient, using another transformer with 12 layers and 12 multi-heads.

The results are provided in Table S5. Although the model showed no performance drop in the external validation (AUC of 0.697 [95% CI, 0.631-0.762]) compared with the internal validation (AUC of 0.708 [95% CI, 0.647-0.769], the diagnostic performances were lower than those of the frame-level diagnosis, probability due to the lack of diversity in data for the patient-level.

**Figure S1. Study Flow Diagram**


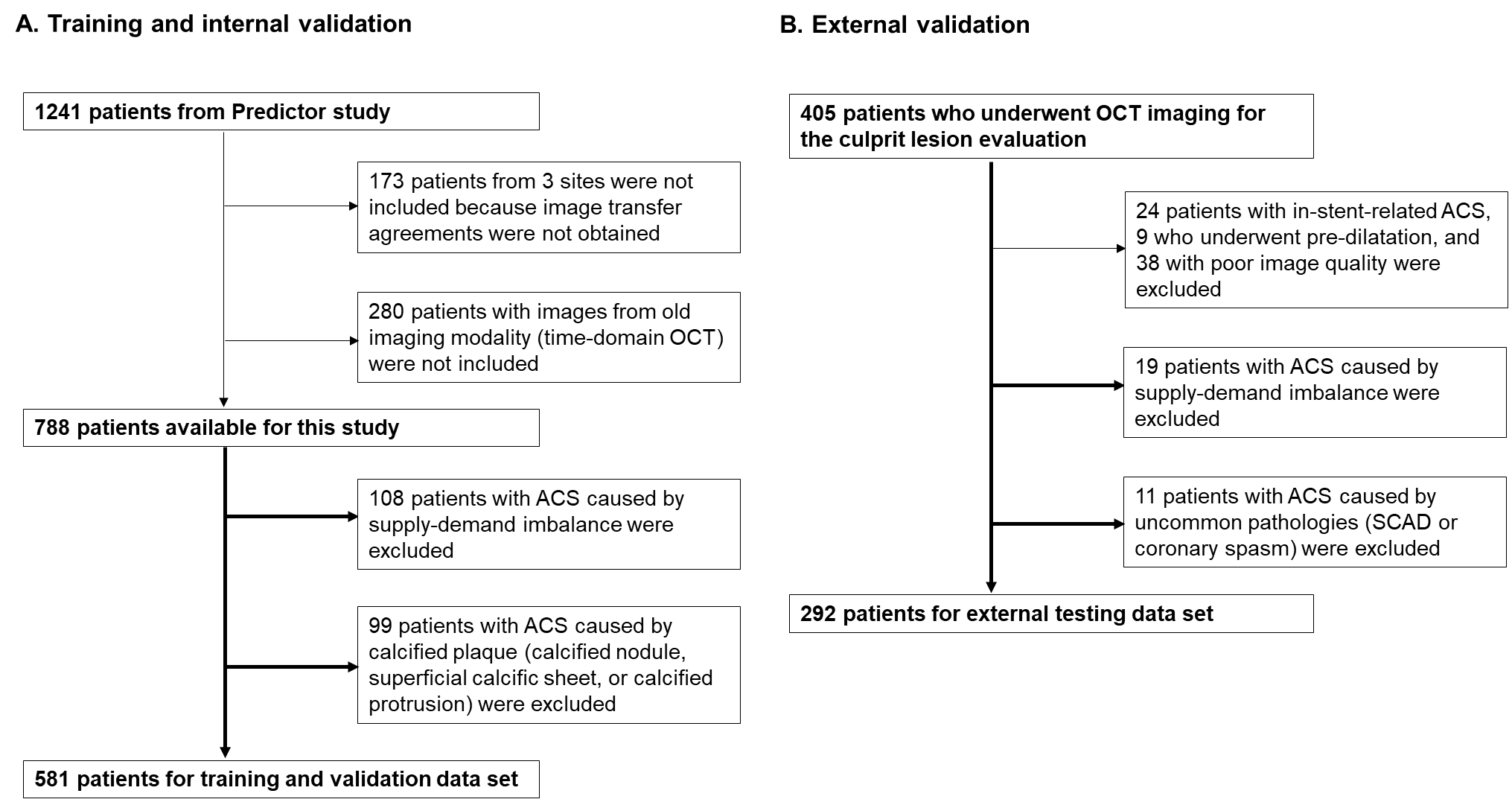


(A) After excluding ineligible patients, 581 patients from the Identification of Predictors for Coronary Plaque Erosion in Patients with Acute Coronary Syndrome (Predictor) study were used for the training and internal validation dataset. (B) To evaluate the developed model, 292 patients from the Effective Anti-Thrombotic Therapy Without Stenting: Intravascular Optical Coherence Tomography–Based Management in Plaque Erosion (EROSION) study were used as an external validation dataset. OCT=optical coherence tomography. ACS=acute coronary syndrome. SCAD=spontaneous coronary artery dissection.

**Figure S2. Architecture of the deep learning models**


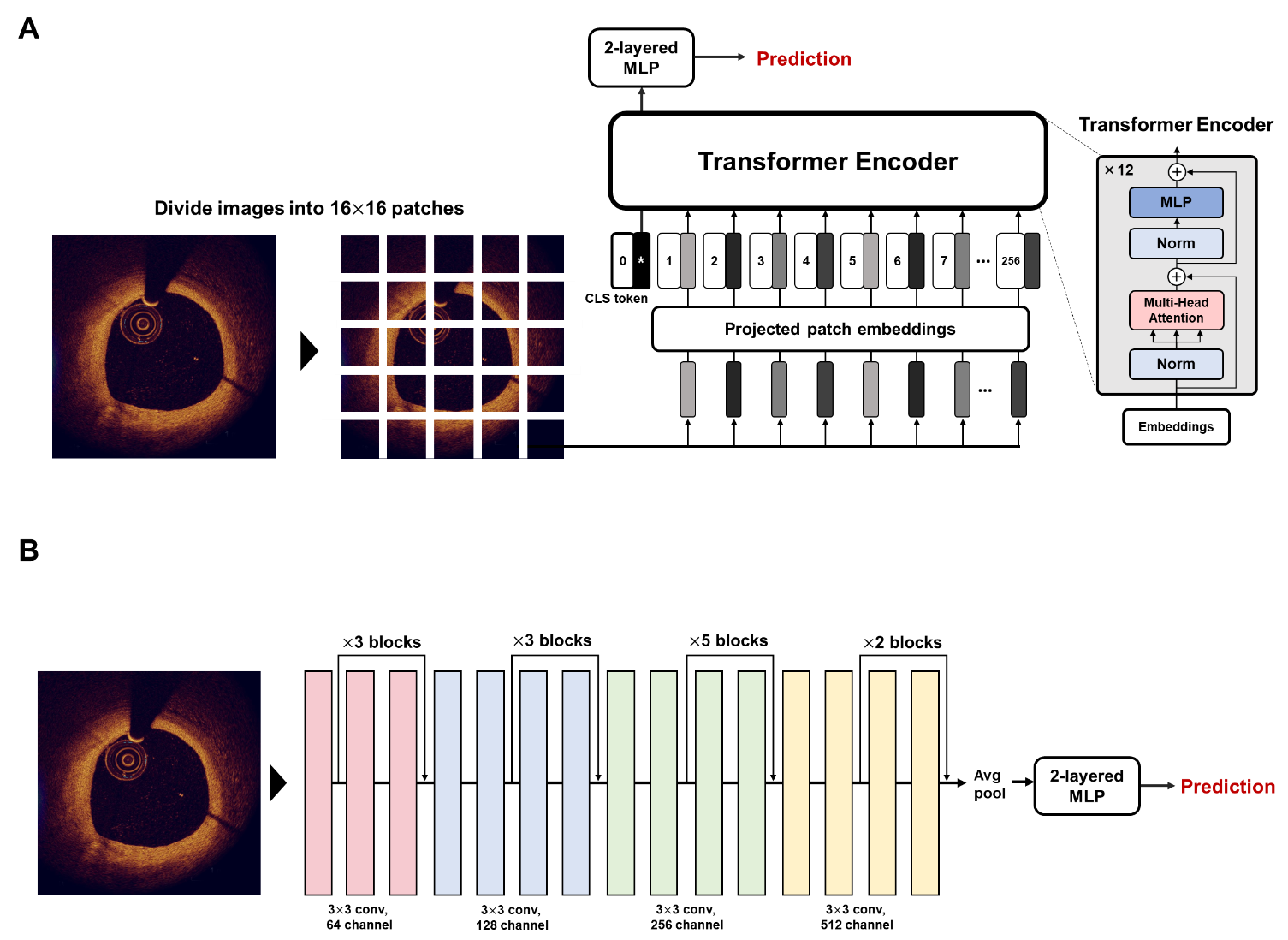


(A) Details of the model architecture for the vision transformer (ViT)-based model. We implemented the ViT-S16-based model for this study. (B) Details of the model architecture for convolutional neural network (CNN)-based model. The ResNet-34 model was used as the standard CNN-based model.

**Figure S3. Details of attention visualization methods**


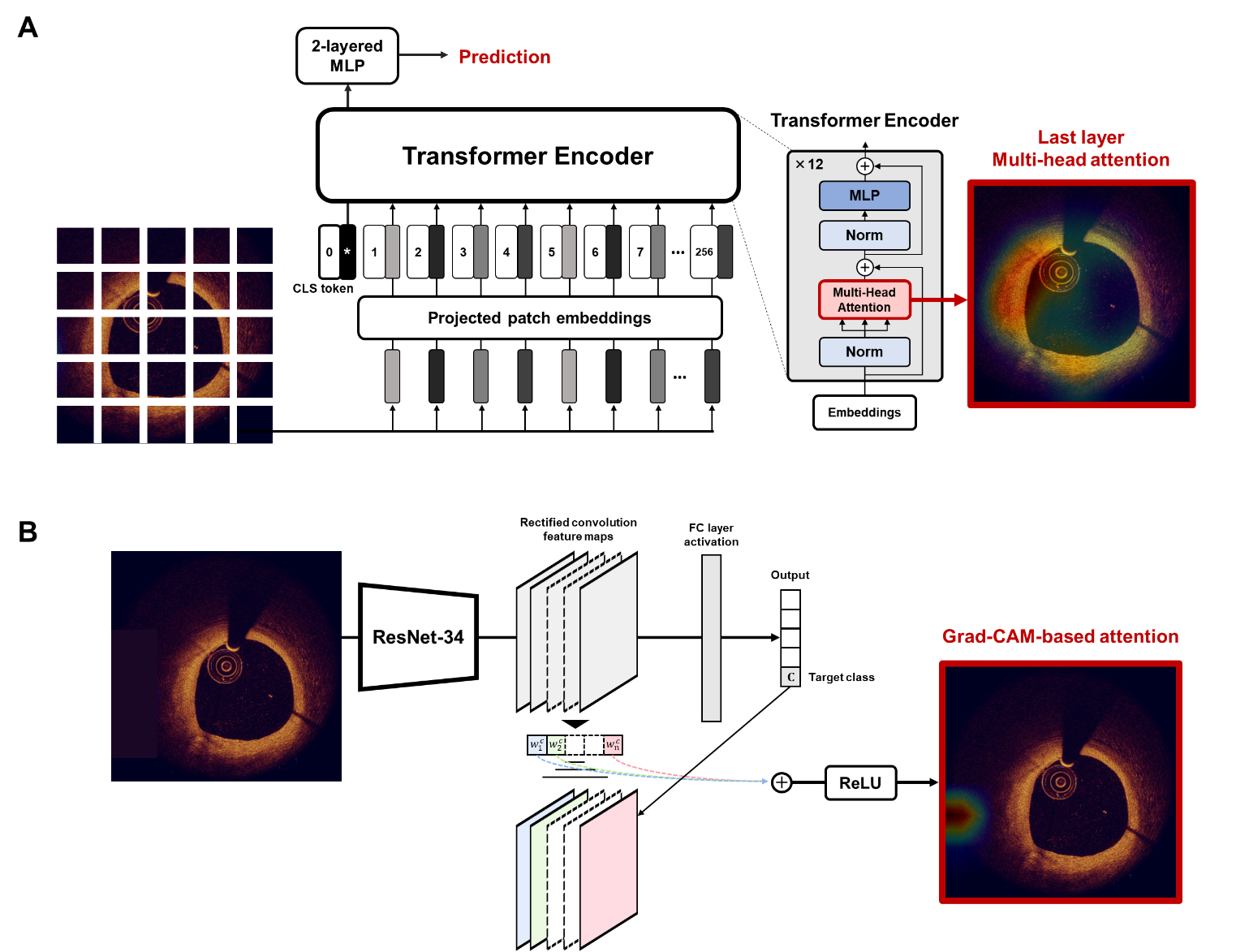


(A) Details of the attention visualization method for vision transformer (ViT)-based model. Since the ViT model utilizes self-attention within the transformer encoder, the multi-head attention was directly visualized. On the other hand, (B) indirect visualization of the important area with gradient-weighted class activation map (Grad-CAM) was used for the standard convolutional neural network-based model, since it does not utilize any direct attention mechanism within it.

**Figure S4. Details of the multi-frame ensemble method and the representative cases of correction**


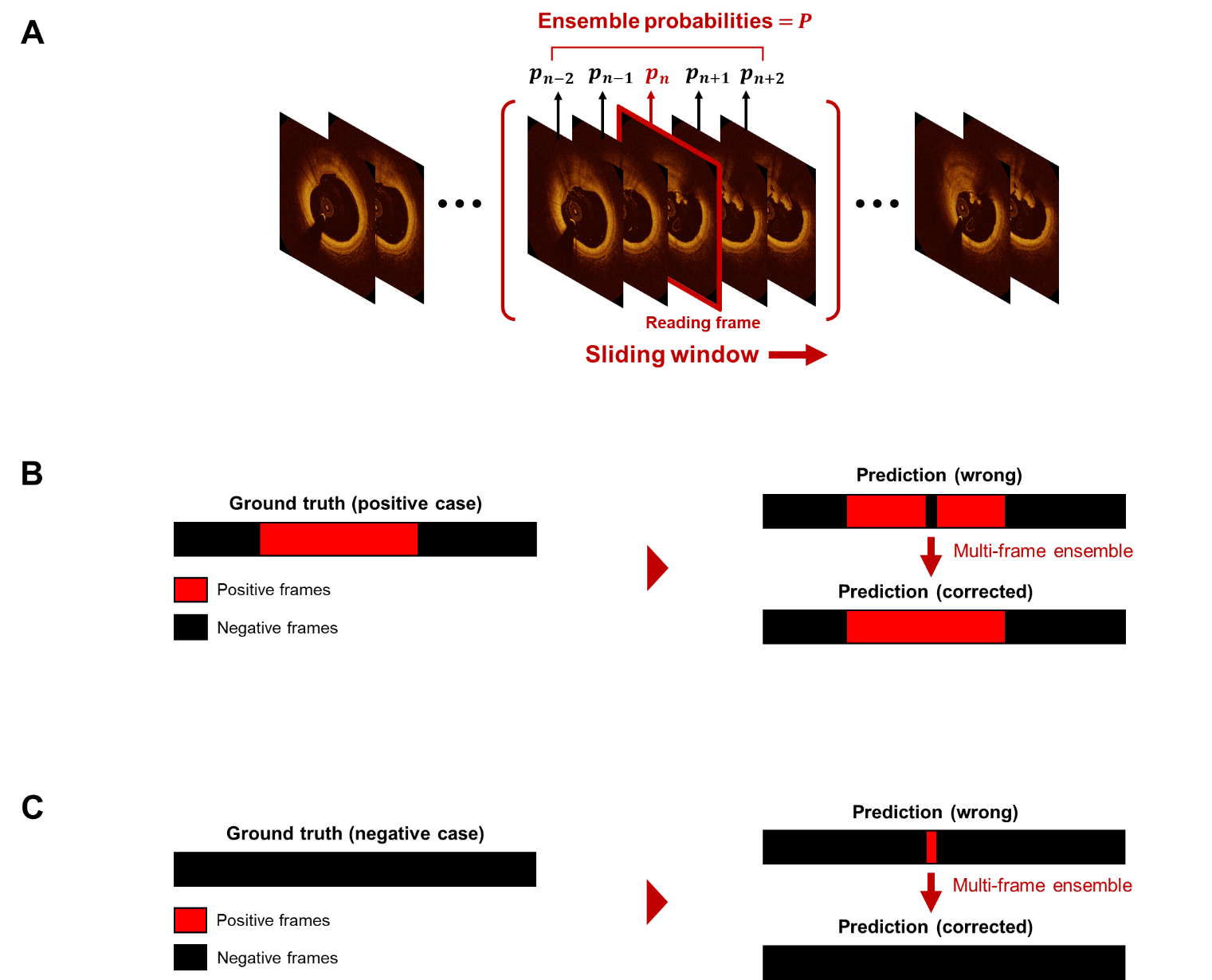


(A) Details of the multi-frame ensemble of adjacent frames (window size = 5), which resembles the recognition process of the experienced OCT reader. Thanks to this ensemble, the mistaken predictions in either (B) positive case or (C) negative case could be properly corrected.

**Figure S5. Deep learning model for the patient-level diagnosis.**


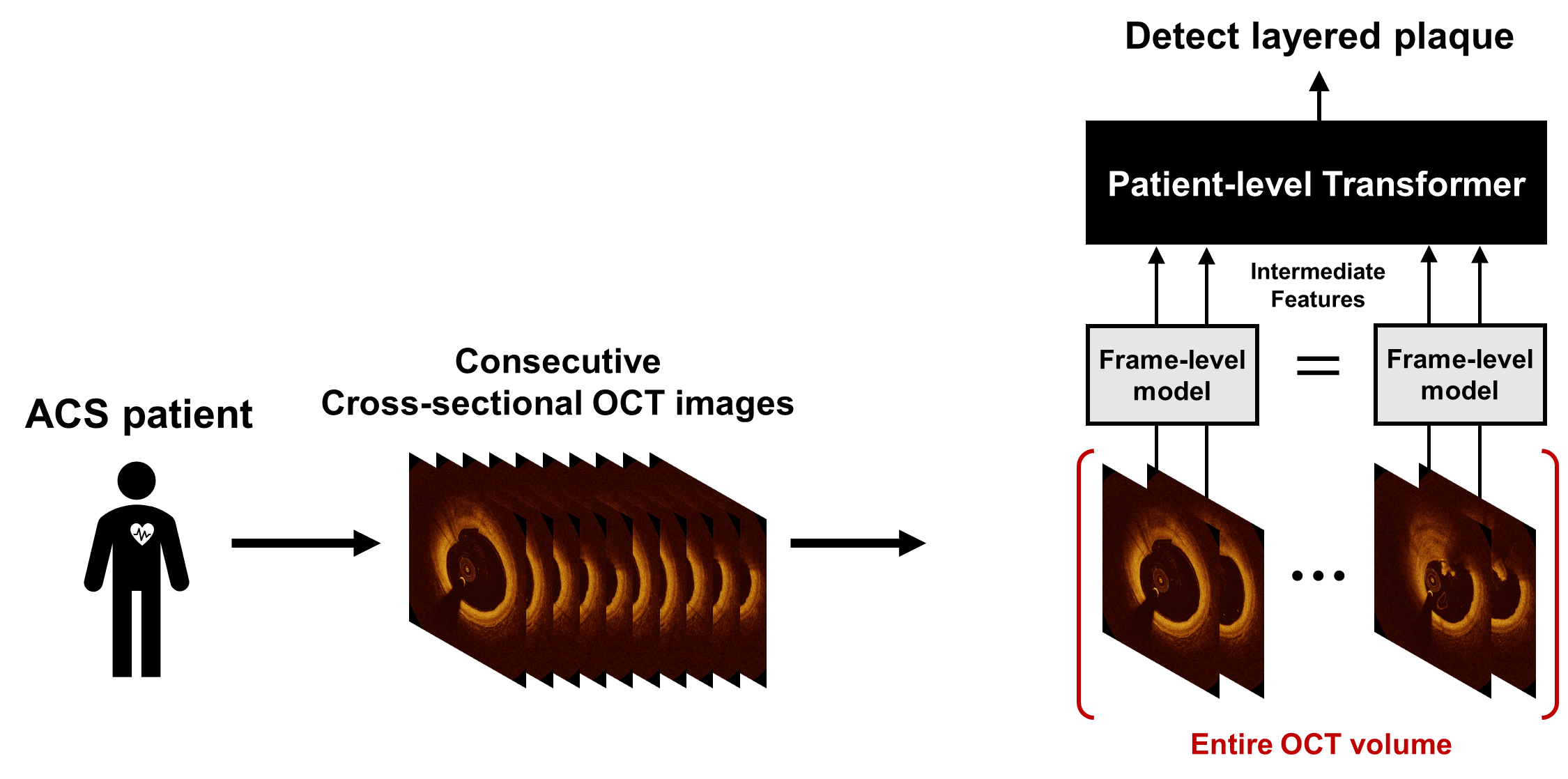

The model integrates the frame-level features and makes the diagnosis for the entire optical coherence tomography (OCT) volumes of each patient. ACS, acute coronary syndrome.

**Table S1. Summary of the Countries and Institutions of the Predictor Study**

| **Participating sites** | **Country** | **Number of included patients** |
| --- | --- | --- |
| Nara Medical University Hospital | Japan | 150 |
| Nippon Medical Chiba Hokusoh Hospital | Japan | 109 |
| Hirosaki University Hospital | Japan | 88 |
| Massachusetts General Hospital | US | 72 |
| The Chinese University of Hong Kong | Hong Kong | 61 |
| Tsuchiura Kyodo General Hospital | Japan | 59 |
| Kitasato University Hospital | Japan | 22 |
| University Hospitals Leuven | Belgium | 20 |
| Total |  | 581 |

**Table S2. Summary of hyperparameters of the deep learning models**

| **Hyperparameters** | **Value** |
| --- | --- |
| *ViT-based model* |  |
| Input image size | 224 × 224 |
| Batch size | 360 |
| Learning rate | 0.000001 |
| Learning rate scheduler | OneCycleLR |
| Optimizer | AdamW |
| Epochs | 20 |
| Image feature dimension | 384 |
| Weight decay coefficient | 0.1 |
| Model architecture | ViT-S16 |
| Number of transformer encoder | 12 |
| Number of multi-head per layer | 6 |
| Image patch size | 16 |
| *CNN-based model* |  |
| Input image size | 224 × 224 |
| Batch size | 360 |
| Learning rate | 0.000001 |
| Learning rate scheduler | OneCycleLR |
| Optimizer | AdamW |
| Epochs | 20 |
| Image feature dimension | 512 |
| Weight decay coefficient | 0.1 |
| Model architecture | ResNet-34 |
| ViT, vision transformer; CNN, convolutional neural network; OneCycleLR, one-cycle learning rate. | |

| **Table S3. Performance of the ViT-based model with and without multi-frame ensemble for frame-level diagnosis in the internal and external validation** | | | | | | |
| --- | --- | --- | --- | --- | --- | --- |
|  | **AUC (95% CI)** | **Sensitivity (%) (95% CI)** | **Specificity (%) (95% CI)** | | **Accuracy (%) (95% CI)** |  |
| **Internal validation** | | | | | | |
| No ensemble | 0.851 (0.846 - 0.857) | 75.4 (74.0 - 76.8) | 78.2 (78.0 - 78.3) | 78.1 (77.9 - 78.3) | |  |
| Ensemble | 0.860 (0.855 - 0.866) | 77.7 (76.4 – 79.0) | 77.6 (77.4 – 77.8) | 77.6 (77.4 – 77.8) | |  |
| **External validation** | | | | | | |
| No ensemble | 0.833 (0.824 - 0.841) | 72.5 (70.5 - 74.5) | 76.9 (76.6 - 77.2) | 76.8 (76.4 - 77.1) | |  |
| Ensemble | 0.845 (0.837 - 0.853) | 76.5 (74.6 – 78.4) | 76.0 (75.7 – 76.3) | 76.0 (75.7 – 76.3) | |  |
| CNN, convolutional neural network; AUC, area under the curve; CI, confidence interval; PPV, positive predictive value; NPV, negative predictive value | | | | | |  |

| **Table S4. Performance of the CNN-based model with and without multi-frame ensemble for frame-level diagnosis in the internal and external validation** | | | | | | |
| --- | --- | --- | --- | --- | --- | --- |
|  | **AUC (95% CI)** | **Sensitivity (%) (95% CI)** | **Specificity (%) (95% CI)** | | **Accuracy (%) (95% CI)** |  |
| **Internal validation** | | | | | | |
| No ensemble | 0.790 (0.783-0.797) | 70.3 (68.9-71.8) | 73.8 (73.6-74.0) | 73.7 (73.6-73.9) | |  |
| Ensemble | 0.799 (0.792-0.805) | 71.7 (70.2-74.0) | 73.8 (73.6-74.0) | 73.8 (73.6-73.9) | |  |
| **External validation** | | | | | | |
| No ensemble | 0.781 (0.772-0.791) | 70.1 (68.0-72.1) | 71.9 (71.5-72.2) | 71.8 (71.5-72.1) | |  |
| Ensemble | 0.791 (0.782-0.800) | 71.4 (69.3-73.4) | 71.9 (71.6-72.3) | 71.9 (71.6-72.2) | |  |
| CNN, convolutional neural network; AUC, area under the curve; CI, confidence interval; PPV, positive predictive value; NPV, negative predictive value | | | | | |  |

| **Table S5. Performance of the deep learning models for patient-level diagnosis in the internal and external validation** | | | | |
| --- | --- | --- | --- | --- |
|  | **AUC (95% CI)** | **Sensitivity (%) (95% CI)** | **Specificity (%) (95% CI)** | **Accuracy (%) (95% CI)** |
| **Patient-level model** | | | | |
| Internal validation | 0.697 (0.631-0.762) | 67.1 (55.8-77.1) | 66.7 (62.3-70.8) | 66.7 (62.7-70.6) |
| External validation | 0.708 (0.647-0.769) | 67.9 (58.2-76.7) | 64.5 (57.2-71.4) | 65.8 (60.0-71.2) |
| AUC, area under the curve; CI, confidence interval; PPV, positive predictive value; NPV, negative predictive value; DL, deep learning; ViT, vision transformer; CNN, convolutional neural network. | | | | |

**References**

1. Dosovitskiy A, Beyer L, Kolesnikov A et al. An image is worth 16x16 words: Transformers for image recognition at scale. arXiv preprint arXiv:201011929 2020.

2. Deng J, Dong W, Socher R, Li L-J, Li K, Fei-Fei L. Imagenet: A large-scale hierarchical image database. 2009 IEEE conference on computer vision and pattern recognition: Ieee, 2009:248-255.

3. Caron M, Touvron H, Misra I et al. Emerging properties in self-supervised vision transformers. arXiv preprint arXiv:210414294 2021.

4. He K, Zhang X, Ren S, Sun J. Deep residual learning for image recognition. Proceedings of the IEEE conference on computer vision and pattern recognition, 2016:770-778.

5. Selvaraju RR, Cogswell M, Das A, Vedantam R, Parikh D, Batra D. Grad-cam: Visual explanations from deep networks via gradient-based localization. Proceedings of the IEEE international conference on computer vision, 2017:618-626.
